# Aspirin eugenol ester regulates cecal contents metabolomic profile and microbiota in an animal model of hyperlipidemia

**DOI:** 10.1186/s12917-018-1711-x

**Published:** 2018-12-18

**Authors:** Ning Ma, Xi-Wang Liu, Xiao-Jun Kong, Shi-Hong Li, Zeng-Hua Jiao, Zhe Qin, Ya-Jun Yang, Jian-Yong Li

**Affiliations:** 1 Key Lab of New Animal Drug Project of Gansu Province; Key Lab of Veterinary Pharmaceutical Development, Ministry of Agriculture, Lanzhou Institute of Husbandry and Pharmaceutical Science of Chinese Academy of Agricultural Sciences, No.335, Jiangouyan, Qilihe district, Lanzhou, 730050 China; 2 College of Veterinary Medicine, Agricultural University of Hebei, Baoding, Hebei 071000 China

**Keywords:** Aspirin eugenol ester, Gut microbiota, Metabonomics, Cecal contents, Hyperlipidemia, UPLC-Q-TOF/MS, High fat diet

## Abstract

**Background:**

Hyperlipidemia, with an increasing of prevalence, has become one of the common metabolic diseases in companion animal clinic. Aspirin eugenol ester (AEE) is a novel compound that exhibits efficacious anti-hyperlipidemia activities. However, its mechanisms are still not completely known. The objective of present study was to investigate the intervention effects of AEE on cecal contents metabonomics profile and microbiota in hyperlipidemia rats.

**Results:**

Three groups of rats were fed with a control diet, or high fat diet (HFD) containing or not AEE. The results showed the beneficial effects of AEE in HFD-fed rats such as the reducing of aspartate aminotransferase (AST) and total cholesterol (TCH). Distinct changes in metabonomics profile of cecal contents were observed among control, model and AEE groups. HFD-induced alterations of eight metabolites in cecal contents mainly related with purine metabolism, linoleic acid metabolism, glycerophospholipid metabolism, sphingolipid metabolism and pyrimidine metabolism were reversed by AEE treatment. Principal coordinate analysis (PCoA) and cluster analysis of microbiota showed altered patterns with distinct differences in AEE group versus model group, indicating that AEE treatment improved the negative effects caused by HFD on cecal microbiota. In addition, the correction analysis revealed the possible link between the identified metabolites and cecal microbiota.

**Conclusions:**

This study showed regulation effects of AEE on cecal contents metabonomics profile and microbiota, which could provide information to reveal the possible underlying mechanism of AEE on hyperlipidemia treatment.

**Electronic supplementary material:**

The online version of this article (10.1186/s12917-018-1711-x) contains supplementary material, which is available to authorized users.

## Background

As an emerging discipline, metabonomics provide a powerful approach to discover biomarkers in biological systems [1]. Based on the measurement of global metabolite profiles, metabonomics has been increasingly applied to investigate the responses of living systems to genetic modifications or pathophysiological stimuli. At present, liquid chromatography-mass spectrometry (LC-MS) has become one of the frequently used techniques in metabonomics studies for its numerous advantages such as high sensitivity and reproducibility [2]. Gut microbiota is now considered as a vital factor for human health and disease [3]. It has been recognized that gut microbiota plays important roles in many key functions of the host, which are associated with reproduction, obesity, cancer, nutrition restriction and gut immune maturation [4, 5].

Hyperlipidemia has now become a serious health issue in human and companion pets such as dogs and cats [6, 7]. In animals, as a health risk factor, hyperlipidemia is involved in the progress of many diseases such as inflammation, diabetes mellitus, obesity, atherosclerosis and hypertension. Many studies suggest that the disorder of lipid metabolism is one of the main features of hyperlipidemia, which can lead the abnormal levels of triglycerides (TG), total cholesterol (TCH), low-density lipoprotein (LDL) and high-density lipoprotein (HDL). In the market, many drugs such as statins, nicotinic acid and fibrates are commonly used for hyperlipidemia treatment. But unfortunately, some side or toxic effects of these drugs, for instance, statin-induced myopathy and fibrate-induced rhabdomyolysis, have limited their clinical application [8]. Therefore, to develop a safe and effective drug for hyperlipidemia treatment is becoming a research hotspot in the world.

As we all known, aspirin is widely used for the treatment of inflammation, fever, arthritis and the prevention of cardiovascular disease. Moreover, some studies indicate that aspirin has therapeutic effects on dyslipidemia and related diseases [9]. A number of reports have demonstrated that eugenol has remarkable anti-hyperlipidemia effect such as the improvement of abnormal lipid profiles in rats fed with high fat diet (HFD) [10, 11]. However, the side effects such as gastrointestinal damage of aspirin and vulnerability of eugenol limit their application. These disadvantages are mainly caused by the carboxyl group of aspirin and hydroxyl group of eugenol. In order to reduce side effect and improve stabilization through chemical structural modification, aspirin eugenol ester (AEE), a pale yellow and odourless crystal, was synthesized with the starting precursors of aspirin and eugenol according to the pro-drug principle [12]. Many studies including toxicity, teratogenicity, metabolism, pharmacodynamics and stability of AEE have been carried out in our lab, and the results indicate that AEE is a promising compound with good druggability [13–15].

We previously established the hyperlipidemia model in rats induced by HFD, and investigated the regulation effects of AEE on blood lipids [16, 17]. Moreover, the effects of AEE on the metabonomics profiles of plasma, urine, liver and feces were also explored [18, 19]. The results indicated that AEE was an effective compound for hyperlipidemia treatment, and its mechanism could be partly revealed by the metabonomics study. As important biological samples, the relative abundances of many metabolites in cecal contents are different from those in the feces, which are attractive for biomarker investigation to illustrate the therapeutic basis of drug [20, 21]. It is well known that the concentration and diversity of the microbial communities depend on the sample used [22]. Some studies showed that the bacterial diversity, richness and community composition of fecal samples were low compared to the cecal contents [23]. Little information is known concerning the alteration of cecal contents metabonomics and microbiota associated with AEE therapeutic effects. With the application of ultra performance liquid chromatography-quadrupole time-of-flight mass spectrometry (UPLC-Q-TOF/MS) analysis and 16S rRNA Illumina sequencing, the objective of this follow-up study was to investigate the effects of AEE on cecal contents metabonomics profile and microbiota and find out more evidences to understand the possible underlying mechanism of AEE against hyperlipidemia.

## Results

### Body growth, liver weight and food consumption

**Fig. 1:**
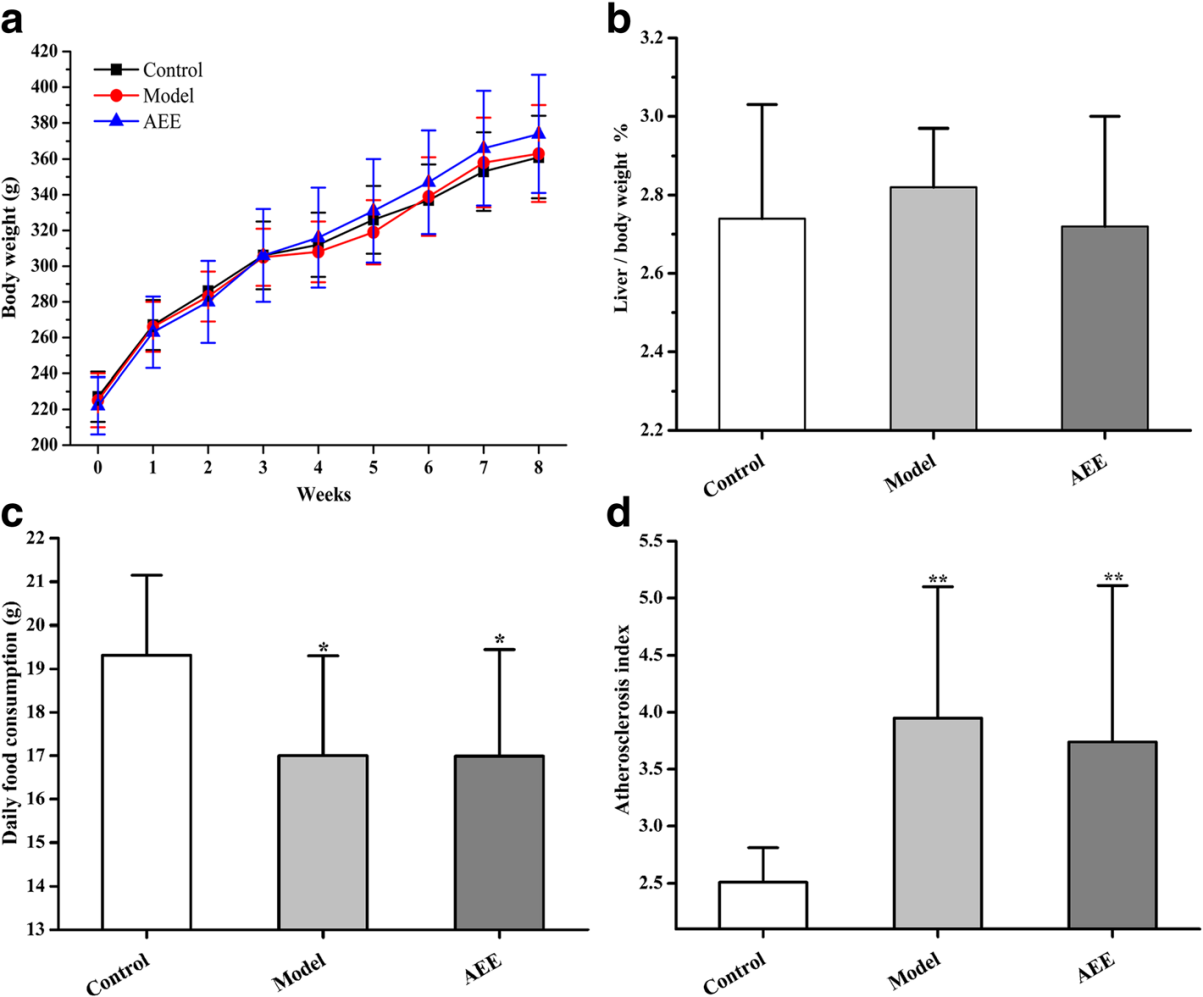
Effects of AEE on hyperlipidemia related indices in rats (*n* = 10). **a** Effects of AEE on body weight. **b** Effects of AEE on liver weight. **c** Effects of AEE on daily food consumption. **d** Effects of AEE on atherosclerosis index. *P < 0.05, **P < 0.01 compared with the control group

There were no differences in the initial or final body weights or liver/body weight ratio among control, model and AEE groups (Fig. 1a and b). Notably, the mean value of liver/body weight ratio in the model group was higher than those in the control and AEE group, but not significant (Fig. 1b). Daily food consumption in the model and AEE groups were significantly decreased (*P* < 0.05) compared to the control (Fig. 1c). There was no statistical difference in food consumption between model group and AEE group (*P* > 0.05).

### Hematological and serum biochemical parameters

Effect of AEE on hematological parameters was shown in Table 1. No significant differences were observed in hematological parameters except platelet (PLT) index. PLT was significantly higher in model group in comparison with the control group (*P* < 0.01), as well as in AEE group (*P* < 0.05). Compared with the model group, AEE treatment reduced the increase of PLT, but there was no significance in statistics.

**Table 1:** Hematological findings in rats fed with HFD supplemented with AEE

Variables	Units	Control	Model	AEE
WBC	10^9^/L	82.1 ± 8.4	87.7 ± 9.4	84.6 ± 6.9
LY	10^9^/L	6.77 ± 1.27	7.74 ± 1.82	7.08 ± 1.10
MONO	10^9^/L	1.84 ± 0.30	2.05 ± 0.35	1.93 ± 0.22
NEUT	10^9^/L	73.7 ± 7.0	77.9 ± 7.5	75.6 ± 5.7
RBC	10^12^/L	9.24 ± 0.96	8.91 ± 0.58	9.16 ± 0.92
PLT	10^9^/L	1034 ± 172	1271 ± 220^**^	1107 ± 143^*^
HCT	%	51.4 ± 5.4	48.9 ± 4.2	50.5 ± 5.0
MCV	fL	55.8 ± 2.6	54.9 ± 2.1	55.2 ± 2.5
RDW-CV	%	14.3 ± 0.6	14.8 ± 0.7	14.6 ± 0.8
MPV	fL	5.73 ± 0.37	5.76 ± 0.42	5.52 ± 0.28
PDW	%	16.3 ± 0.2	16.4 ± 0.3	16.2 ± 0.2

Values were expressed as mean ± SD. ^*^P < 0.05, ^**^P < 0.01 compared with the control group

When compared with the control rats, the results of biochemical parameters indicated that HFD showed strong effects on increasing alanine aminotransferase (ALT), alkaline phosphatase (ALP), TCH, TG and LDL levels, and reducing direct bilirubin (DB) and urea (Table 2, *P* < 0.01). ALP and ALT levels were significantly higher in the AEE group than those in the control group (*P* < 0.01), whereas the creatine kinase (CK), lactate dehydrogenase (LDH), DB, urea and HDL was significantly reduced (*P* < 0.05 and *P* < 0.01). In comparison with the model group, aspartate aminotransferase (AST), LDH and TCH levels in AEE group were significantly reduced (*P* < 0.05 and *P* < 0.01), indicating the partial improvement of biochemical profile in HFD-fed rats. Atherosclerosis index (AI) values in model and AEE groups were significantly increased in comparison with the control (*P* < 0.01, Fig. 1d). No statistical difference of AI was observed between model and AEE groups.

**Table 2:** Serum levels of biochemical parameters of rats in different group

Variables	Units	Control	Model	AEE
TB	μmol /L	1.45 ± 0.28	1.46 ± 0.24	1.38 ± 0.23
DB	μmol /L	1.32 ± 0.47	0.96 ± 0.23^**^	0.93 ± 0.21^**^
TP	g/L	56.8 ± 4.5	56.9 ± 3.5	57.2 ± 3.7
ALB	g/L	35.2 ± 3.0	34.3 ± 1.7	34.7 ± 2.0
GLOB	g/L	21.7 ± 1.8	22.6 ± 2.3	22.5 ± 2.1
ALT	U/L	36.1 ± 3.5	48.2 ± 8.5^**^	50.3 ± 7.3^**^
AST	U/L	125.3 ± 18.1	134.6 ± 29.3	116.7 ± 16.1^#^
ALP	U/L	108.3 ± 19.4	152.2 ± 24.2^**^	156.2 ± 27.3^**^
LDH	U/L	1037 ± 175	1145 ± 267	853 ± 152^*#^
CK	U/L	819 ± 163	913 ± 328	694 ± 148^*^
Urea	mmol/L	7.66 ± 0.68	5.58 ± 0.60^**^	5.68 ± 0.87^**^
CREA	μmol /L	41.8 ± 8.5	40.2 ± 5.4	41.5 ± 4.1
GLU	mmol/L	6.56 ± 1.26	6.94 ± 1.32	7.35 ± 1.21
TG	mmol/L	1.10 ± 0.30	1.41 ± 0.19^**^	1.27 ± 0.22
TCH	mmol/L	1.20 ± 0.08	1.44 ± 0.17^**^	1.29 ± 0.08^##^
HDL	mmol/L	0.46 ± 0.05	0.42 ± 0.05	0.42 ± 0.12
LDL	mmol/L	0.37 ± 0.05	0.44 ± 0.05^**^	0.40 ± 0.03

Values were expressed as mean ± SD. ^*^P < 0.05, ^**^P < 0.01 compared with the control group. ^#^*P* < 0.05, ^##^*P* < 0.01 compared with the model group

### Cecal contents metabolic profiling

**Fig. 2:**
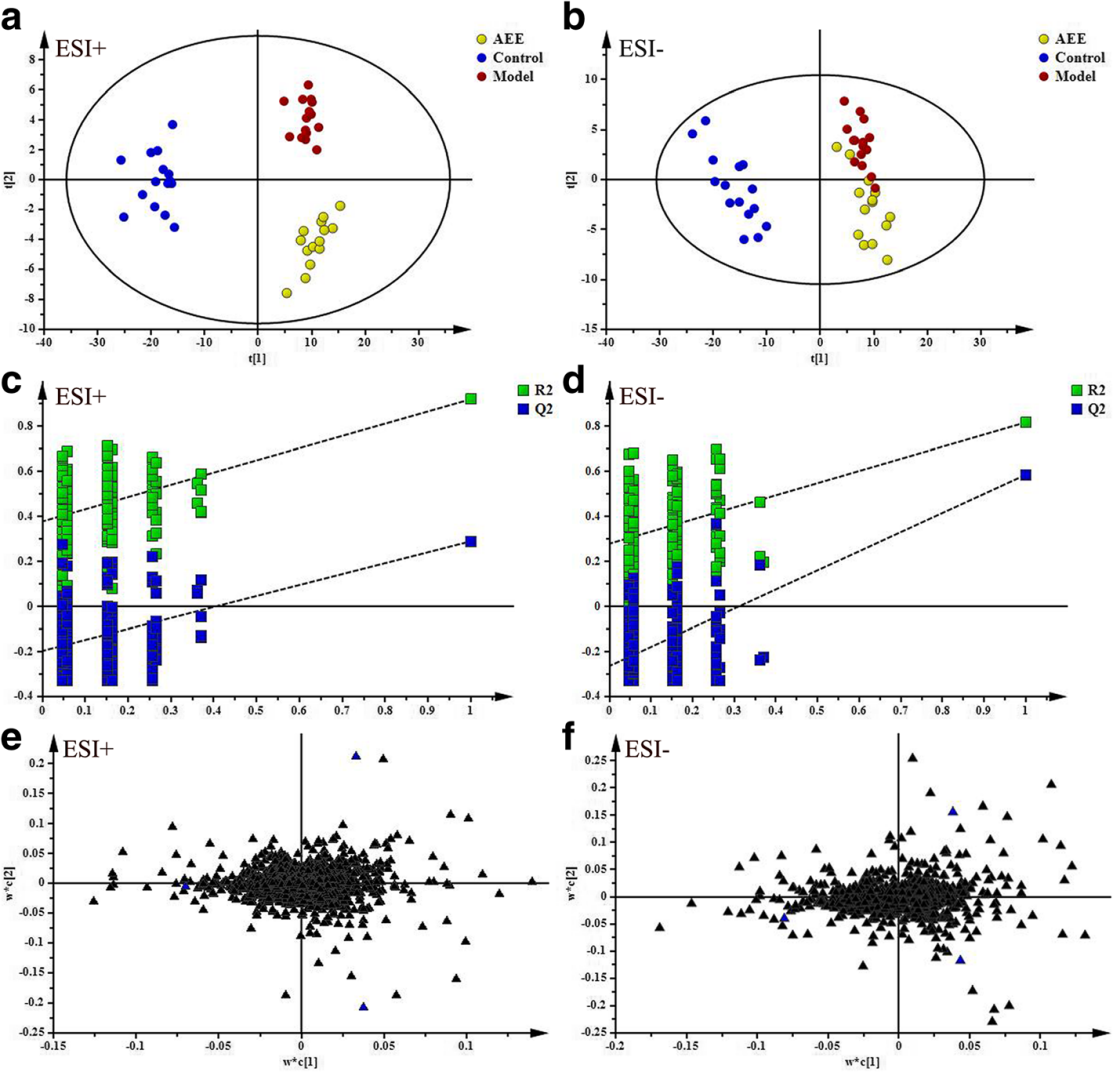
Multivariate data analyses of cecal contents based on UPLC-Q-TOF/MS analysis. ESI+: electrospray ionization in positive ion mode; ESI-: electrospray ionization in negative ion mode. **a** and **b** PLS-DA score plots in positive and negative modes. ESI+: R^2^X = 0.441, R^2^Y = 0.937, Q^2^ = 0.497; ESI-: R^2^X = 0.502, R^2^Y = 0.874, Q^2^ = 0.674. **c** and **d** Plots of the permutation test of the PLS-DA models. ESI+: R^2^ = 0.375, Q^2^ = − 0.211; ESI-: R^2^ = 0.272, Q^2^ = − 0.279. **e** and **f** Loading plots of the PLS-DA models

In this study, an UPLC-Q/TOF MS-based cecal contents metabonomics study was carried out in rats fed with HFD. Representative total ion chromatograms (TICs) of the cecal contents in positive and negative modes were shown in Additional file 1: Figure S1, which displayed good separation effect and strong sensitivity of the established method. Unsupervised principal component analysis (PCA) approach was used to get an overview of the data and monitor the stability of the study (Additional file 1: Figure S2). The PCA score plots showed all quality control (QC) samples were clustered tightly together in positive and negative modes indicating the reliability of the present study. Typically,a well-fitting partial least squares discriminant analysis (PLS-DA) model was constructed to identify and reveal the differential metabolites among control, model and AEE groups. The parameters of PLS-DA models including R^2^X = 0.441, R^2^Y = 0.937 and Q^2^ = 0.497 for positive data, and R^2^X = 0.502, R^2^Y = 0.874, Q^2^ = 0.674 for negative data were obtained. Score plots of PLS-DA models were shown in Fig. 2a and b. In both of positive and negative modes, a clear separation of samples from control and model groups was observed, which indicated remarkable changes in cecal contents induced by HFD. Score plots showed that samples in AEE group were located far away from those in the model group. The results of PLS-DA score plots indicated that AEE treatment partly restored the alterations in cecal contents induced by HFD. The permutation test was applied to guard against overfitting of the PLS-DA models. Validation with 200 random permutation tests generated intercepts of R^2^ = 0.375 and Q^2^ = − 0.211 from positive model data (Fig. 2c) and R^2^ = 0.272 and Q^2^ = − 0.279 from negative model data (Fig. 2d), which demonstrated that the PLS-DA models were robust without overfitting.

### Differential metabolites in cecal contents

In PLS-DA models, loading-plot as a tool was used to identify the metabolites contributing to group separation. As shown in Fig. 2e and f, ions in loading-plot away from center were considered as potential biomarkers responsible group separation. With variance importance for projection (VIP) values above 1 and adjusted *P*-values less than 0.05, 8 metabolites were filtered and identified as potential biomarkers (Table 3). Compared with the control rats, HFD significantly increased the relative intensities on some potential biomarkers including lysophosphatidylcholine (LysoPC) (18:1(9Z)), linoleic acid, linoleoyl ethanolamide, oleamide and sphingosine, and the biomarkers like hypoxanthine, uridine and sebacic acid were significantly reduced (*P* < 0.05 and *P* < 0.01). Notably, AEE treatment partly reversed the abnormal metabolite changes in cecal contents induced by HFD such as the significant reduction of LysoPC (18:1(9Z)) and sphingosine. The pathway results from KEGG revealed that the disturbed pathways in cecal contents were purine metabolism, linoleic acid metabolism, glycerophospholipid metabolism, sphingolipid metabolism and pyrimidine metabolism.

**Table 3:** Potential biomarkers in cecal contents based on the UPLC-Q-TOF/MS analysis and the changes between different groups

SM	RT	VIP	Metabolite	Formula	m/z	Adduction	Fold change		Pathway
							M/C	AEE/M	
+	2.26	2.92	Hypoxanthine	C_5_H_4_N_4_O	137.0459	[M + H]^+^	0.39^**^	1.12	Purine metabolism
+	18.11	2.04	Linoleic acid	C_18_H_32_O_2_	281.2479	[M + H]+	3.78^**^	0.81	Linoleic acid metabolism
+	17.84	1.40	LysoPC(18:1(9Z))	C_26_H_52_NO_7_P	522.3606	[M + H]+	1.90^*^	0.47^*^	Glycerophospholipid metabolism
+	19.32	1.01	Linoleoyl ethanolamide	C_20_H_37_NO_2_	324.2905	[M + H]+	1.59^*^	0.88	–
+	21.26	2.51	Oleamide	C_18_H_35_NO	282.2796	[M + H]+	2.19^*^	0.49^*^	–
+	14.81	8.12	Sphingosine	C_18_H_37_NO_2_	300.2904	[M + H]+	2.98^**^	0.52^**^	Sphingolipid metabolism
–	2.99	1.07	Uridine	C_9_H_12_N_2_O_6_	243.0622	[M-H]-	0.35^**^	1.14	Pyrimidine metabolism
–	10.40	1.17	Sebacic acid	C_10_H_18_O_4_	201.1132	[M-H]-	0.49^*^	1.43	–

*SM* scan model, *RT* retention time, *VIP* Variance importance for projection, *LysoPC*, Lysophosphatidylcholine, *M/C*: model versus control, AEE/M: AEE versus model, ^*^P < 0.05, ^**^P < 0.01 compared with corresponding group

### Effects of AEE on cecal microbiota composition

The effects of AEE on cecal microbiota composition were evaluated by Illumina sequencing. A total of 45 cecal contents were collected and sent for sequencing. However, 4 samples were outliers, and not be used in the further analysis. There were total 3,009,532 qualified reads and an average of 73,403 ± 13,104 reads for each sample. After operational taxonomic units (OUT) picking and chimera checking, the effective reads were generated and assigned to 29,532 non-singleton OTUs. Each sample had 71,884 reads and 720 OTUs on average (Additional file 1: Table S2). Rarefaction analysis was employed to evaluate sequencing depth of each sample, and the results suggested that sufficient sequencing sampling reads could perform a meaningful analysis (Additional file 1: Figure S3).

The effects of HFD and AEE on bacteria community composition at phylum level was shown in Additional file 1: Table S3. The top 10 taxa with high relative abundance were calculated and analyzed. As expected, after being fed with HFD in model and AEE groups, the relative abundances of *Euryarchaeota, Actinobacteria, Tenericutes* and *Saccharibacteria* were decreased and that of *Firmicutes* was increased (*P* < 0.05 and *P* < 0.01). AEE had some reversal effects on microbiota disturbance induced by HFD such as the reduction of *Firmicutes* and the increase of *Euryarchaeota*, but no statistical difference in taxa abundances was observed between AEE and model groups.

At the genus level, ten key microbial genera associated with AEE treatment in the rats fed with HFD were found (Table 4). These genera were selected based on relative abundance and statistical difference, which were responsible for the difference among three groups. Interestingly, significant differences in the cecal contents microbiota of rats fed with HFD were observed. For example, when compared with the control rats, *Corynebacterium_1*, *Nosocomiicoccus*, and *Jeotgalicoccus* were significantly decreased in the rats fed with HFD (*P* < 0.01). A similar decrease of these generas was also observed in AEE group in comparison with the model group (*P* < 0.01). Notably, rats fed with HFD had higher levels of *Turicibacter* and *Bifidobacterium* compared with the control (*P* < 0.01). Remarkable increase of *Bifidobacterium* and reduction of *Turicibacter* were found in AEE group than those in the model (*P* < 0.05). In addition, there was no difference in the relative abundance of *Staphylococcus*, *[Eubacterium]_brachy_group*, *[Ruminococcus]_gauvreauii_group*, *Ruminococcaceae_NK4A214_group* and *[Eubacterium]_xylanophilum_group* between control and model groups. However, AEE had significant influence on these generas such as the increase of *[Ruminococcus]_gauvreauii_group* and the reduction of *Staphylococcus*.

**Table 4:** The relative abundance of key different genera in rats

Genus	Control	Model	AEE
*Staphylococcus*	8.05 ± 5.17	8.19 ± 7.26	2.94 ± 1.45^**##^
*Turicibacter*	8.95 ± 2.98	13.78 ± 5.36^**^	9.46 ± 3.03^#^
*Corynebacterium_1*	2.05 ± 1.63	0.62 ± 0.48^**^	0.23 ± 0.16^**##^
*Bifidobacterium*	0.13 ± 0.09	0.43 ± 0.31^**^	1.29 ± 1.02^**#^
*[Ruminococcus]_gauvreauii_group*	0.40 ± 0.21	0.43 ± 0.15	0.95 ± 0.56^##^
*Nosocomiicoccus*	1.26 ± 0.48	0.42 ± 0.12^**^	0.18 ± 0.13^**##^
*Ruminococcaceae_NK4A214_group*	0.76 ± 0.31	0.79 ± 0.23	1.27 ± 0.38^##^
*[Eubacterium]_xylanophilum_group*	0.77 ± 0.26	0.93 ± 0.35	1.27 ± 0.24^##^
*Jeotgalicoccus*	0.40 ± 0.22	0.16 ± 0.06^**^	0.06 ± 0.05^**##^
*[Eubacterium]_brachy_group*	0.16 ± 0.03	0.17 ± 0.04	0.13 ± 0.03^##^

Data were expressed as (mean ± SD)%. ^*^P < 0.05, ^**^P < 0.01 compared with the control group; ^#^*P* < 0.05, ^##^*P* < 0.01 compared with the model group

### AEE altered cecal microbiota structure

**Fig. 3:**
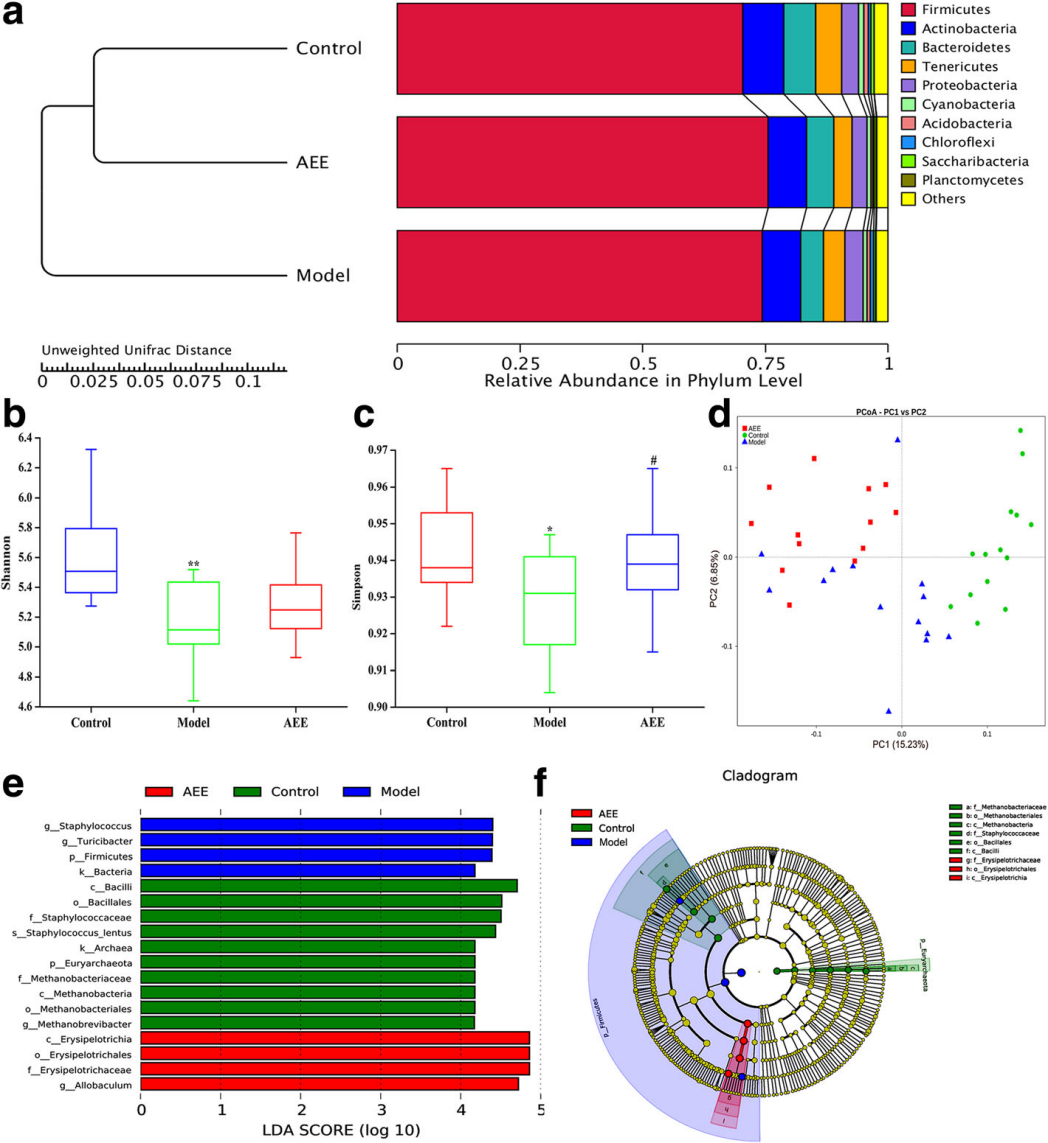
AEE reversed the dysbiosis of caecal microbiota in HFD-induced hyperlipidemia rats. **a** Cluster analysis of the samples based on UPGMA. **b** and **c** Community diversity of each group reflected by Shannon and Simpson indexes with Wilcoxon analysis. ^*^P < 0.05, ^**^P < 0.01, compared with the control group; ^#^*P* < 0.05 compared with the model group. **d** Principal coordinate analysis (PCoA) of bacterial community structures of the gut microbiota of each group. **e** LDA scores as calculated by LEfSe analysis. Only taxa with LDA scores of more than 4 were presented. **f** LEfSe cladogram representing different abundant taxa

A phylogenic tree analysis based on the unweighted pair-group method with arithmetic mean (UPGMA) was used to cluster the cecal samples in different group. Figure 3a showed that samples in control and AEE groups were grouped closely, and samples in model group were branched separately. These results suggested that the microbial communities in AEE groups were more similar to the control than model. Next, changes in microbial communities were investigated using alpha diversity measures including Shannon’s diversity index and Simpson (estimated OTUs) (Fig. 3b and c). Significant differences were found between control and model groups. HFD had highly significant effects to reduce both Shannon and Simpson diversity indexes (*P* < 0.01 and *P* < 0.05), showing that the diversity of the cecal microbiota were significantly decreased in rats with feeding HFD. In regard to Shannon diversity index, AEE increased HFD-reduced diversity and reduced the difference, but there was no significant difference between model and AEE groups. Interestingly, the Simpson index, indicating the community richness, was also increased in AEE groups, and significant difference was found between model and AEE groups (*P* < 0.05). These data suggested that HFD decreased the abundance and diversity of the cecal microbiota in rats, while AEE treatment ameliorated them.

Unweighted unifrac distance based principal coordinates analysis (PCoA) was used to examine the relationship of the community structures. The PCoA plots (sample’s microbiota represented by symbol) revealed a distinct clustering of microbiota composition for each treatment group (Fig. 3d). The microbiotas of the model group were distinct from those in control, indicating that HFD had significant impact on the microbiota community. Meanwhile, samples in AEE groups were also significantly separated from those in model group. Notably, 7 samples of cecal contents in AEE group were clustered together near the control suggesting the improvement of HFD-induced microbiota dysbiosis.

In order to study the difference of the cecal microbiota, a linear discriminant analysis effect size (LEfSe) with LDA score at least 4 was performed. *Archaea, Euryarchaeota* and *Methanobrevibacter* were found more in the control group (Fig. 3e). Greater proportions of *Turicibacter and Staphylococcus* were enriched in the model than in the control (Fig. 3e). The taxonomic abundances in the cecal microbiota of the model and AEE groups were also compared with LEfSe analysis. Figure 3e showed that bacteria taxa such as *Allobaculum* and *Erysipelotrichaceae* were different between model and AEE groups. LEfSe cladogram of bacterial lineages was used to provide an easily appreciated view of the enrichment profiles for each group (Fig. 3f). At a phylum level, cecal microbiota of HFD-feeding rats was enriched with *Firmicutes*, suggesting that HFD had an impact on these bacteria. Similarly, the phylum *Firmicutes* were also enriched in AEE treated rats. Additionally, it was observed that *Euryarchaeota* was enriched in rats from the control group.

### Correlation between cecal microbiota and metabolites

**Fig. 4:**
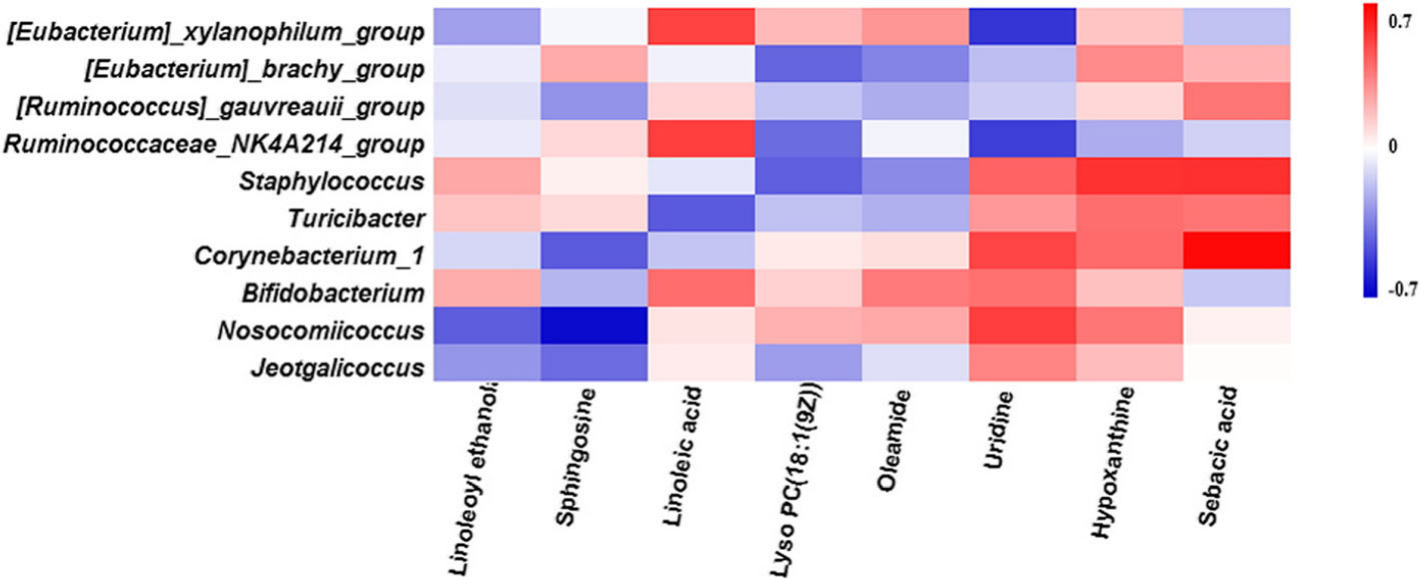
Pearson correlation between caecal microbiota (at the genera level) and potential biomarker affected by AEE treatment. Red color indicating positive correlations whereas blue denoting negative correlations

Correlation between cecal contents metabolites and microbiota in the rats from AEE group was also investigated in present study. Pearson correlation was analyzed between the selected metabolites and the cecal microbiota abundance at the genus level. Interestingly, a clear correlation with the metabolites in cecal contents was found for the disturbed cecal microbiota at genus level. In Fig. 4, the red color indicated positive correlations between metabolites and generas, whereas blue denoted the negative correlations. Linoleoyl ethanol and sphingosine showed negative correction with *g__Nosocomiicoccus* and *g__Jeotgalicoccus*. Five generas (*Staphylococcus*, *Turicibacter*, *Jeotgalicoccus*, *Corynebacterium_1*, *Bifidobacterium* and *Nosocomiicoccus*) showed positive correlation with uridine and hypoxanthine, but *Ruminococcaceae_NK4A214_group* and *[Eubacterium]_xylanophilum_group* showed negative correlation with uridine and linoleic acid. Sebacic acid was positively correlated with *Staphylococcus* and *Corynebacterium_1.* However, sebacic acid showed no correlation with *Nosocomiicoccus* and *Jeotgalicoccus*, and a similar result was observed among linoleic acid, *Nosocomiicoccus*, *Jeotgalicoccus* and *[Eubacterium]_brachy_group*.

## Discussion

As one of the most common metabolic disease, pathophysiology of hyperlipidemia is very complex and has been only partially elucidated. Previous study reported that the development of hyperlipidemia was accompanied with the changes of gut microbiota and metabonomics profile [24]. AEE consists of two chemical structural units from aspirin and eugenol. Pharmacokinetics results showed that AEE was directly decomposed into salicylic acid and eugenol after oral administration, which could exhibit their original activities and act synergistically [13]. In this work, metabonomic analysis and cecal microbiota were applied to systemically evaluate the treatment effects of AEE in hyperlipidemia rats. The results showed that HFD consumption induced significant changes in cecal contents metabolic profiles and microbiota, whereas AEE could reverse the HFD-induced these alterations in hyperlipidemia rats. This study indicated that the integration of metabonomics study and gut microbiota is a sensitive and effective approach in drug development. Moreover, the results of this study could provide additional evidence to illustrate the possible action mechanism of AEE against hyperlipidemia.

Both of aspirin and eugenol have good effects on improving hyperlipidemia induced by HFD [25–27]. Therefore, as the combination of aspirin and eugenol, AEE has stronger therapeutic efficiency than its precursors on hyperlipidemia treatment [16]. In our previous study, serum biochemical results had confirmed that AEE could significantly reduce the levels of TG, LDL and TCH in hyperlipidemic rats induced by the HFD [16, 17]. In present study, the consumption of HFD caused the disorder of lipid profiles such as the increase of TG, TCH and LDL. These results indicated that the hyperlipidemia disease model was established successfully. AEE ameliorated the HFD-induced blood lipid disorder such as the significant reduced levels of TCH, and low values of LDL and TG, which were consistent with the previous results [17]. However, it was noted that the regulation effects of AEE on blood lipid profile in present study were weaker than those in our previous results [16–18]. Evidences from numerous studies have shown that the same drug with different administration way can produce various effects through the changes in pharmacokinetic profiles [28, 29]. In our previous studies, AEE suspension was prepared in 0.5% sodium carboxymethyl cellulose, and the method of gavage administration was performed in rats. In present study, AEE was added in the HFD, and then be eaten by rats. Therefore, it was speculated that the pharmacokinetic differences caused by drug delivery way were the possible reasons for the weak regulation effects of AEE on blood lipids in this study. In addition, the weak effects of AEE might be attributable to the dosage used in this study that AEE dosage (43.5 mg/kg) was lower than the optimal AEE dosage (54 mg/kg) for hyperlipidemia treatment found in our previous research [16].

AI is a reliable index to access the lipid contribution to the cardiovascular disease risk. Elevated AI values in the model group were mainly associated with the HFD-induced lipid orders. However, AEE showed no influence on AI index in present study, which was inconsistent with the previous results found in atherosclerotic hamster [30]. There are several possible reasons for these results. First, different administration method used in the experiment might be the main reason for the poor regulation effects on AI index. Second, the differences of HFD composition, animals and the duration of the experiment may be the other reasons for the AI results. Literature indicated that the lipid metabolism and cholesterol transport of rat and hamster are different [31]. There was no difference in daily food consumption between model and AEE groups. Therefore, the improved lipid profile in AEE group was not related to the food rejection. It was also noticed that there was no difference in body weight between control and model groups, which might be related with inadequate HFD consumption [32].

ALT and AST are important enzymes in the liver, which can be served as indicators of liver function. The mean serum levels of ALT and AST showed an increase in model group after HFD feeding for 8 weeks compared with those in the control group. These changes of ALT and AST showed that the rats should had developed liver function damage. After administration of AEE, the serum AST was significantly reduced, indicating that AEE had positive effect on liver function. Some researchers have reported that the liver weight is increased after the rats fed with HFD, which is related with the pathological changes in the liver such as the edema and steatosis [33]. In present study, although the liver weight was increased in the model group, there was no statistical difference among control, model and AEE groups. It is worth noting that increasing studies have showed that lipid disorders could cause decline of renal function caused by oxidative or pathological damages [34, 35]. In present study, CREA showed no difference and urea was significantly reduced in model and AEE groups. Urea is the principal end product in the metabolism of nitrogen-containing compounds in animals, especially for protein. Percentage of protein in the HFD (18.3%) was lower than that in the standard diet (24.4%), which might be the reasons for the reduced urea levels in model and AEE groups. It was observed that there was a significant increase in platelet of the rats in the model group, which was in agreement with the previous study [36]. Evidence from numerous studies has shown that there is a close relationship between lipid disorder and platelet, for instance, HFD-induced dyslipidemia could lead platelet adhesion and aggregation. [37] Administration of AEE decreased the mean level of platelet, which might attribute to the ameliorative effects on lipid profiles.

Metabonomics is a sensitive and powerful tool to provide quantitative measures of global changes in the metabolic profile. UPLC-Q-TOF/MS analysis method was used in present study, in conjunction with multivariate data analysis, to identify the metabolites significantly affected by AEE treatment in cecal contents. The metabolomic analysis indicated that there was a significant difference in metabolic patterns of the control, model and AEE groups in the score plots. The results of metabonomics study were partly in agreement with the findings in blood biochemistry and cecal microbiota, indicating the improvement of AEE on hyperlipidemia and the interactions among blood lipid, metabonomics and microbiota. Moreover, 8 metabolites (e.g. hypoxanthine, linoleic acid, sphingosine, uridine, sebacic acid) were selected as potential biomarkers which were associated with sphingolipid metabolism, purine metabolism, linoleic acid metabolism, glycerophospholipid metabolism and pyrimidine metabolism.

Sphingosine is a primary part of sphingolipids, and can be phosphorylated to the formation of sphingosine-1-phosphate. Several publications have demonstrated that increased dietary saturated fat content can elevate sphingolipid metabolism, and the abnormal sphingolipid metabolism is closely associated with obesity and hyperlipidemia [38]. In addition, the inhibitation of sphingolipid metabolism could improve circulating lipids through the reduction of LDL [39]. In our study, sphingosine was increased in hyperlipidemic rats, which was matched with other reports that sphingosine was enhanced in the hamster fed with HFD [40]. Notably, AEE treatment showed favorable inhibition on sphingosine, suggesting that the depression of AEE on sphingolipid metabolism might contribute to its efficacy on hyperlipidemia.

The disturbance of glycerophospholipid and fatty acid metabolism is found to be directly associated with the initiation and progression of hyperlipidemia. In this study, the alterations of potential biomarkers including LysoPC (18:1(9Z)), linoleic acid and oleamide had influence on the metabolism of glycerophospholipid and fatty acid. Linoleic acid, a carboxylic acid, is a polyunsaturated omega-6 fatty acid. Oleamide is an amide of the fatty acid oleic acid and the substrates of fatty acids amide hydrolase. In present study, linoleic acid and oleamide in cecal contents were increased in the hyperlipidemic rats compared with the control rats. These results suggested that the fatty acids oxidation of hyperlipidemic rats was blocked, which could accumulate fatty acid level and cause dyslipidemia [41]. In contrast to the model, the levels of oleamide and linoleic acid were reduced after AEE treatment, which implied that AEE could improve lipid disorders by regulating fatty acid metabolism. LysoPCs, served as precise marker for specific metabolic disease, play important roles in the development of cardiovascular disease by triggering inflammation and the autoimmune response [42]. LysoPC (18:1(9Z)) was increased in the model group, suggesting the glycerophospholipid metabolism was promoted under hyperlipidemia condition. This increase could be significantly inhibited by AEE treatment, implying that the therapeutic effect of AEE on hyperlipidemia might ascribe to the inhibition of glycerophospholipid metabolism. Sebacic acid is a dicarboxylic acid with 10 carbon atoms, which can produce important intermediates of energy metabolism such as acetyl-CoA and succinyl-CoA. Some researchers have reported that sebacic acid was increased in the feces in atherosclerotic rats [43]. Inconsistent with the abovementioned studies, the content of sebacic acid was reduced in the model group, indicating that the HFD might destroy the equilibrium of energy metabolism. AEE treatment could inhibit the down-regulation of sebacic acid, suggesting AEE could ameliorate the disturbed energy metabolism.

Metabolites such as uridine and hypoxanthine related to purine and pyrimidine metabolism were also identified in the study. Uridine and hypoxanthine are pyrimidine and purine derivatives, respectively. Several recent publications have demonstrated that hypoxanthine and uridine could be significantly reduced in the liver of the obese mice under HFD [44], and the hypoxanthine in the feces was also decreased by the microfloral population reduction [45]. Consistent with the above results, levels of uridine and hypoxanthine were lower in the model group than those in the control, indicating that HFD intake could lead the suppression of purine metabolism and pyrimidine metabolism, or the reduction of gut microbiota. AEE treated group showed recovery patterns of hypoxanthine and uridine. From these results, HFD might induce alterations in the metabolisms of purines and pyrimidine or gut microbiota, which could be attenuated by the AEE treatment. Linoleoyl ethanolamide was a fatty acid ethanolamide. There is no study about the relationship of linoleoyl ethanolamide with hyperlipidemia, and it would be interesting to investigate the biological function of linoleoyl ethanolamide in hyperlipidemic rats in future studies.

Compared with our previous fecal metabonomics study, lower number of metabolites was found in the cecal contents in present study [19]. Poor therapeutic effects caused by the administration method might have limited impact on the cecal contents metabolites, which might be the reason for finding few metabolites. Polakof et al reported that linoleic acid level in the cecal contents from HFD-fed rats was significantly higher than the control rats, which was consistent with the changes of linoleic acid in present study [46]. Surprisingly, opposite change trends of linoleic acid were observed in previous fecal metabonomics studies [19]. It has been reported that oxidation products major from linoleic acid in feces were significantly increased in the HFD-induced atherosclerotic rats [43]. Oxidation process of linoleic acid might severely deplete itself, which could result in low level of linoleic acid in feces. In addition, there was a close relationship between linoleic acid metabolism and bacterial community [47]. Different microbiota composition in cecal contents and feces from control and HFD-fed rats might cause the difference in linoleic acid metabolism. Therefore,it was speculated that linoleic acid had different metabolic transformation process in cecal contents and feces, which might be the reason for heterogeneous results of linoleic acid. In HFD-fed rats, AEE treatment might affect linoleic acid metabolism or microbiota composition to regulate linoleic acid level in cecal contents and feces.

Recently, it has been reported that the bacteria in the gut interact extensively with the host through the metabolic exchange and co-metabolism of substrates and gut bacterial composition was closely linked to hyperlipidemia [4, 48]. In the present study, both PCoA and cluster analysis indicated that AEE treatment altered the structural composition of the cecal microbiota and reversed the dysbiosis caused by HFD. PCoA score plots and cluster analysis of the samples in AEE group showed tendencies similar to that of the control. Yet the microbial community was not completely restored in the rats after AEE treatment in present study. From the view of Shannon and Simpson indexes, AEE displayed positive effects on microbial diversity. The obtained results showed that HFD changed the abundance and diversity of the gut microbiota in rats. For example, the abundance of *Firmicutes* and *Euryarchaeota* increased and decreased, respectively. *Firmicutes* could absorb the calories in the diet and increase the fat storage in the body. Therefore, the improved rats’ blood lipids in AEE treatment group may be related to the recovered abundance and diversity of the gut microbiota such as the reduction of *Firmicutes*.

Combination of LEfSe and statistical significance was employed to determine the features which might explain the differences among groups. Interestingly, the results of cladogram showed that *Methanobacteria* were observed in the control. Previous study proved that *Methanobacteria* can scavenge ammonia as substrates for the generation of methane and to increase the capacity of polysaccharide-metabolizing bacteria [49]. So the residing of *Methanobacteria* in the gut of the rats in the control group was beneficial for physiological functions. Results of LDA score showed a significant increase of phylum *Firmicutes* with major *Turicibacter* in HFD rats as compared to the control. Susanne et al. reported that the relative proportion of *Turicibacter* could be increased by the HFD in the C57BL/6 J mice, and *Turicibacter* had a strong positive correction with body weight gain and energy harvest [50]. Conversely, the relative abundance of *Turicibacter* was lower in the AEE treated rats, which might have beneficial effects on hyperlipidemia treatment by inhibiting energy absorption. In addition, *Staphylococcus* was significantly reduced in the AEE group in comparison with the model. It was reported that *Staphylococcus* infection in HFD-fed dogs could lead to the impairment of glucose tolerance through the damages of insulin secretion and insulin sensitivity [51]. It is known that there is a close relationship between hyperlipidemia and glucose metabolism [52]. Therefore, the reduction of *Staphylococcus* caused by AEE might improve glucose tolerance, which had benefits on hyperlipidemia treatment or reducing diabetes risk to keep host healthy.

Moreover, the correlations were observable between the cecal contents metabolites and microbiota, which could provide interactive functional information associated with AEE treatment. A great number of studies have confirmed the correlations between gut microbiota and metabonomics in HFD-treated animals [24, 53]. Our results showed that there was a possible link between the altered microbiota and metabolites in AEE-treated rats. However, the sophisticated mechanism between endogenous metabolites and microbes affected by AEE treatment has not been clearly elucidated. In Table 4, it was important to note that the effects of AEE treatment on some genera were changed parallel with those in the model group. There were two possible reasons for these results. First, gut microbiota contains some 10^13^–10^14^ bacteria, each of them with their own unique sensitivity to drug. For example, eugenol is known to possess antimicrobial activity in a wide spectrum of bacteria from various levels of concentrations [54, 55]. It was speculated that the AEE treatment had diverse effects on gut bacteria such as bacteriostatic or bactericidal effects, which might result in the changes of genera in gut microbiota. Second, the different changes of endogenous metabolites may be other potential cause. Increasing evidences have indicated the significant interplay between gut microbiota and mammalian metabolism. In this study, AEE made a significant difference on cecal contents metabolites, and then changed metabolites might affect genera abundance. In future, more studies are needed to elucidate the interactions between AEE and specific genera.

It was important to note that this study had some limitations. First, this study did not compare the effects of AEE with its parent compounds, which could provide direct evidence to display the advantages of AEE. Second, the deep action mechanism among AEE, microbiota and metabolic pathways remains unknown. Further studies are needed to be done to explicate the interactions of AEE with microbiota and metabolic pathways associated with hyperlipidemia.

## Conclusions

LC–MS based metabolomics and 16S rRNA gene sequencing were combined to assess the effects of AEE on HFD-induced hyperlipidemia. The results showed that AEE treatment ameliorated not only cecal contents metabolism but also cecal microbiota composition in HFD-fed rats. The metabonomic analysis revealed that eight metabolites involved in purine metabolism, linoleic acid metabolism, glycerophospholipid metabolism, sphingolipid metabolism and pyrimidine metabolism were regulated by AEE treatment. AEE also normalized the HFD-induced alternations in the gut microbiota such as the reduction of *Staphylococcus* and *Turicibacter.* Furthermore, the correlation analysis revealed the possible link between the identified metabolites and gut microbiota. These findings indicated the regulation effects of AEE on cecal contents metabonomics profile and microbiota, which could provide new evidence to understand the possible action mechanism of AEE for hyperlipidemia treatment.

## Methods

### Reagents and materials

AEE (transparent crystal, purity: 99.5% with RP-HPLC) was prepared in Key Lab of New Animal Drug Project of Gansu Province, Key Lab of Veterinary Pharmaceutical Development of Agricultural Ministry, Lanzhou Institute of Husbandry and Pharmaceutical Sciences of Chinese Academy of Agricultural Science. The commercial kits for blood biochemical parameters were provided by Ningbo Medical System Biotechnology Co., Ltd. (Ningbo, China). MS-grade formic acid was supplied by TCI (Shanghai, China). Deionized water (18 MΩ) was prepared with a Direct-Q®3 system (Millipore, USA). MS-grade acetonitrile was purchased from Thermo Fisher Scientific (USA). Standard compressed rat feed and high diet feed (HFD) were supplied by Keao Xieli Feed Co., Ltd. (Beijing, China). Standard rat diet consisted of 12.3%lipids, 63.3% carbohydrates, and 24.4% proteins (kcal) and HFD (77.8% standard diet, 10% yolk power, 10% lard, 2% cholesterol and 0.2% bile salts) consisted of 41.5% lipids, 40.2% carbohydrates, and 18.3% proteins (kcal).

### Animals and grouping

**Fig. 5:**
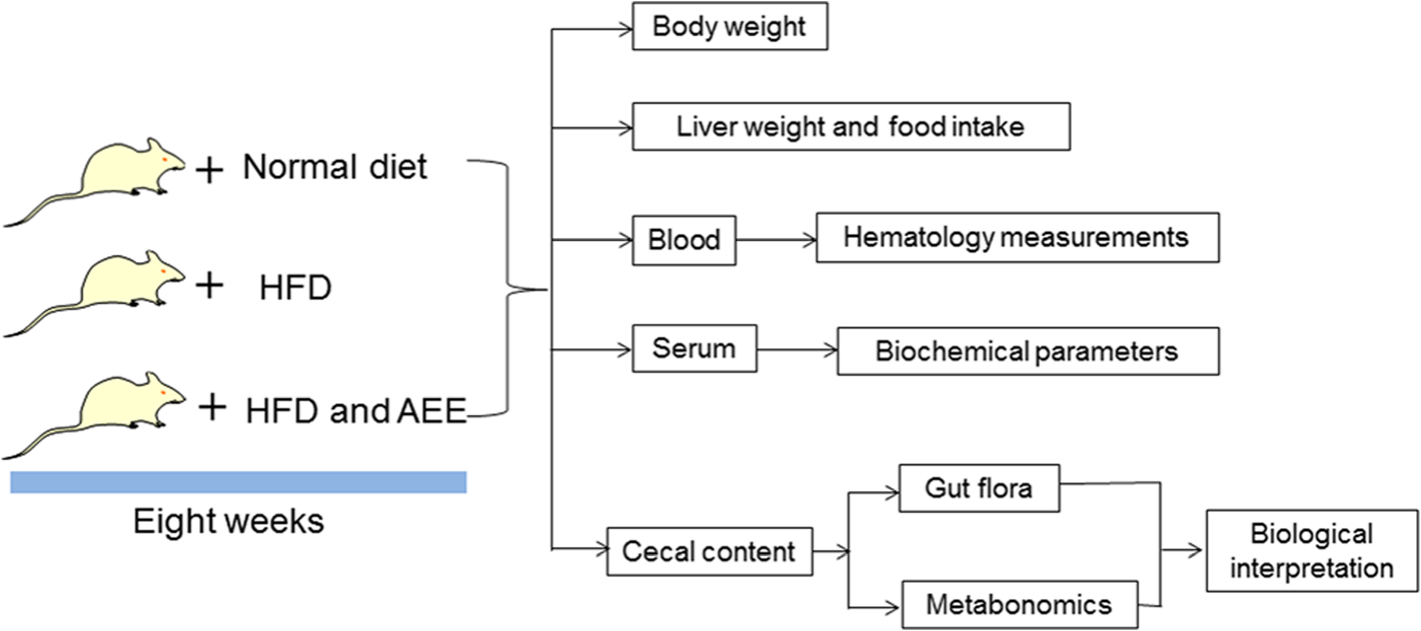
Flowchart of the study design in this experiment

Forty-five male Wistar rats, weighing 180–200 g, were purchased from Lanzhou Veterinary Research Institute (Lanzhou, China). Rats were housed in plastic cages (size: 50 × 35 × 20 cm, 10 rats per cage) with stainless steel wire cover and chopped bedding. Rat feed and drinking water were supplied ad libitum. Light/dark regimen was 12/12 h and living temperature was 22 ± 2 °C with relative humidity of 55 ± 10%. After 2-week adaptation, rats were randomly separated into three groups (15 rats in each group with two cages, 7 or 8 rats per cage) and fed experimental diets for eight weeks. One group as control group was fed with standard diet, whereas the other two groups were fed with a high fat diet containing or not AEE (HFD and HFD plus AEE, respectively). AEE powder was added in HFD at dose of 850 mg/kg diet, and the approximate dose of AEE administered to rats was 43.5 mg/kg body weight in the experiment. The food intake of each group and body weights of individual rats were recorded weekly. A summary of study design used in this work was shown in Fig. 5.

### Sample collection

At the end of experiment, rats were fasted for 10–12 h before blood sampling. Rats were euthanatized with 1% pentobarbital sodium (intraperitoneal injection, 30 mg/kg), and then the blood samples were withdrawn from the heart into different vacuum tubes [56]. The blood in Na-heparin vacuum tubes were used for hematological measurement which was performed in one hour. Blood in vacuum tubes without anticoagulant were centrifuged to obtain serum (4000 *g*, 4 °C for 10 min). Serum samples were stored at − 80 °C until biochemical analysis. Cecal contents were rapidly removed and frozen in liquid nitrogen, and then were stored at − 80 °C until processed.

### Blood analysis

Whole blood was analyzed by BC2800-Vet (Mindray, China) to perform hematological measurements. Hematological parameters were consisted of white blood cell (WBC), lymphocyte (LY), monocyte (MONO), neutrophils (NEUT), red blood cell (RBC), PLT, hematocrit (HCT), mean corpuscular volume (MCV), red blood cell distribution width coefficient of variation (RDW-CV), mean platelet volume (MPV), and platelet distribution width (PDW). With the application of XL-640 automatic analyzer (Erba, Germany), serum samples were analyzed to measure the levels of biochemical parameters including total bilirubin (TB), direct bilirubin (DB), total protein (TP), albumin (ALB), globulin (GLOB), ALT, AST, ALP, LDH, CK, urea, creatinine (CREA), glucose (GLU), TG, HDL, LDL and TCH. For assessing lipid changes, the AI was calculated as followed: AI = (TCH - HDL)/HDL.

### Sample preparation for metabonomics analysis

Cecal contents were lyophilized and then pulverized. 300 μL cold (− 20 °C) methanol was added into 50 mg cecal contents in 2 mL centrifuge tube. After vortex-mixing for 2 min, the mixture was treated with ultrasonic extraction and then centrifuged at 14,000 *g* for 15 min at 4 °C. After the supernatant was filtered through a 0.22 μm nylon filter, an aliquot of 2 μL was injected for analysis. Four QC samples prepared by mixing equal aliquots of cecal contents supernatant were inserted regularly in the analysis sequence.

### Metabolic profiling data acquisition

Chromatographic separation was carried out on an Agilent Eclipse Plus-C18 RRHD column (2.1 × 150 mm, 1.8 μm, Agilent Technologies, USA) using UPLC system consisted of a degasser, thermostat,two binary pumps and autosampler (1290, Agilent Technologies, USA). The column was maintained at 35 °C and eluted at a flowing rate of 0.25 mL/min, using a mobile phase of solvent A - water with 0.1% formic acid (by volume) and solvent B - acetonitrile with 0.1% formic acid (by volume). The optimized gradient program is shown in Additional file 1: Table S1.

Agilent 6530 Q-TOF (Agilent Technologies, USA) was used to perform the mass spectrometry with an electrospray ionization source (ESI). The MS data was collected both in positive and negative ion modes. The fragment voltage was set at 135 V in both modes. The capillary voltages were set at 4.0 KV and 3.5 KV in positive and negative modes, respectively. The desolvation gas rate was set to 10 L/min at 350 °C with the use of drying gas nitrogen. The pressure of the nebulizer was set at 45 psig. Data was collected in centroid mode from 50 to 1000 *m/z* and the scan time was set at 1 spectra/second. MS/MS analysis was carried out to confirm the structure of the potential biomarkers. In addition, biochemical reactions and physiological roles related with endogenous metabolites were searched through KEGG and HMDB.

### Metabonomic data analysis

The raw MS spectra were firstly converted to common data format (.mzData) by Mass Hunter Qualitative Analysis software (Agilent technologies, USA). Then peak alignment was carried out by XCMS program. Subsequently an integrated data matrix composed of compound mass, retention time, and peak intensities was generated. After normalization, the obtained data sets were imported into SIMCA-P V13.0 (Umetrics AB, Sweden) to perform PCA and PLS-DA. In order to avoid over fitting, PLS-DA models were validated by permutation test (with 200 permutations). The parameters of the PLS-DA models including R^2^X, R^2^Y, and Q^2^Y were analyzed to ensure the model quality, and the R^2^Y-, Q^2^Y-intercepts of permutation test were examined and to avoid the risk of over-fitting. VI*P* values and loading-plots were applied to find potential biomarkers. A Wilcoxon Mann Whitney test with false discovery rate (FDR) limit equal to 0.05 was employed for univariate analysis. With VIP value above 1.0 and adjusted *P* value below 0.05, the candidate metabolites were considered to be potential biomarkers.

### DNA extraction and sequencing of cecal microbiota

Total bacteria DNA was extracted from caecal contents by using PowerFecal™ DNA Isolation kit (MO BIO Laboratories, Carlsbad, CA, USA) according to manufacturer’s instruction. The 16S rRNA gene was analyzed to evaluate the bacterial diversity by using Illumina Hiseq (Novogene Bioinformatics Technology Co., Ltd.). 515f/806r primer set targeted the V4 region of the bacterial 16S rDNA was used for DNA amplification. PCR reaction was performed using phusion high-fidelity PCR Mastermix ((New England Biolabs LTD., China) and PCR products were purified by using Qiagen Gel Extraction Kit (Qiagen, Germany). Sequencing libraries were generated using TruSeq® DNA PCR-Free Sample Preparation Kit (Illumina, USA) and index codes were added. The library quality was assessed on the Qubit@ 2.0 Fluorometer (Thermo Scientific) and Agilent Bioanalyzer 2100 system. At last, the library was sequenced on an Illumina HiSeq 2500 platform and 250 bp paired-end reads were generated.

Pairs of reads from the original DNA fragments were merged by using FLASH (V1.2.7, http://ccb.jhu.edu/software/FLASH/). Sequencing reads was assigned to each sample according to the unique barcode of each sample. Chimeric sequences were removed using the USEARCH software and the microbial diversity was analyzed using the QIIME software (Version 1.7.0) with Python scripts (http://qiime.org/). OTUs were picked with a 97% similarity threshold. Alpha diversity and Beta diversity (both weighted and unweighted unifrac) were calculated with QIIME (Version 1.7.0) and displayed with R software (Version 2.15.3). Cluster analysis was preceded by PCoA using WGCNA package, stat packages and ggplot2 package in R software.

### Statistical analysis

The results of the data were expressed as mean ± standard deviation (SD). The differences among experimental groups had been evaluated by one-way ANOVA with Fisher’s least significant difference (LSD) test using the Statistical Package for Social Science program (SPSS 16.0, Chicago, IL, USA). The significance threshold was set at *P* < 0.05 for the test.

## Additional file

Additional file 1: **Table S1.** Optimized gradient elution program of UPLC-Q-TOF/MS in cecal content metabonomic study. **Table S2.** OTU table summary of the samples. **Table S3.** Difference in relative abundanceof gut microbiotaatphylum level. **Figure S1.** TIC of cecal content samples in positive and negative modes. **Figure S2.** PCA score plot basedon the cecal content metabolic profiling in positive and negative modes. **Figure S3.** Rarefactioncurve of the cecal content samples. (PDF 336 kb)

## Abbreviations

AEE: Aspirin eugenol ester
AI: Atherosclerosis index
ALB: Albumin
ALP,: Alkaline phosphatase
ALT: Alanine aminotransferase
AST: Aspartate aminotransferase
CK,: Creatine kinase
CREA: Creatinine
DB: Direct bilirubin
ESI: Electrospray ionization source
FDR: False discovery rate
GLOB: Globulin
GLU: Glucose.
HDL: High-density lipoprotein
HFD: High fat diet
LDH: Lactate dehydrogenase
LDL: Low-density lipoprotein
LysoPC: Lysophosphatidylcholine
OUTs: Operational taxonomic units
PCA: Principal component analysis
PCoA: Principal coordinate analysis
PLS-DA: Partial least squares discriminant analysis
PLT: Platelet QC, quality control
TB: Total bilirubin
TCH: Total cholesterol
TCH: Total cholesterol
TG: Triglycerides
TP: Total protein
VIP: Variance importance for projection

## References

[1] Robertson DG, Frevert U (2013). Metabolomics in drug discovery and development. Clin Pharmacol Ther.

[2] Wilson ID, Plumb R, Granger J, Major H, Williams R, Lenz EM (2005). HPLC-MS-based methods for the study of metabonomics. J Chromatogr B.

[3] Wikoff WR, Anfora AT, Liu J, Schultz PG, Lesley SA, Peters EC, Siuzdak G (2009). Metabolomics analysis reveals large effects of gut microflora on mammalian blood metabolites. Proc Natl Acad Sci U S A.

[4] Zhong Y, Marungruang N, Fak F, Nyman M (2015). Effects of two whole-grain barley varieties on caecal SCFA, gut microbiota and plasma inflammatory markers in rats consuming low- and high-fat diets. Brit J Nutr.

[5] Wang Z, Roberts AB, Buffa JA, Levison BS, Zhu W, Org E, Gu X, Huang Y, Zamanian-Daryoush M, Culley MK (2015). Non-lethal inhibition of gut microbial trimethylamine production for the treatment of atherosclerosis. Cell.

[6] Bowry AD, Lewey J, Dugani SB, Choudhry NK (2015). The burden of cardiovascular disease in low- and middle-income countries: epidemiology and management. Can J Cardiol.

[7] Weeth LP (2016). Other risks/possible benefits of obesity. Vet Clin North Am Small Anim Pract.

[8] Wagstaff LR, Mitton MW, Arvik BM, Doraiswamy PM (2003). Statin-associated memory loss: analysis of 60 case reports and review of the literature. Pharmacotherapy.

[9] Lin HL, Yen HW, Hsieh SL, An LM, Shen KP (2014). Low-dose aspirin ameliorated hyperlipidemia, adhesion molecule, and chemokine production induced by high-fat diet in Sprague-Dawley rats. Drug Dev Res.

[10] Venkadeswaran K, Thomas PA, Geraldine P (2016). An experimental evaluation of the anti-atherogenic potential of the plant, Piper betle, and its active constitutent, eugenol, in rats fed an atherogenic diet. Biomed Pharmacother.

[11] Mnafgui K, Kaanich F, Derbali A, Hamden K, Derbali F, Slama S, Allouche N, Elfeki A (2013). Inhibition of key enzymes related to diabetes and hypertension by eugenol in vitro and in alloxan-induced diabetic rats. Arch Physiol Biochem.

[12] Li J, Yu Y, Wang Q, Zhang J, Yang Y, Li B, Zhou X, Niu J, Wei X, Liu X (2012). Synthesis of aspirin eugenol ester and its biological activity. Med Chem Res.

[13] Shen Y, Liu X, Yang Y, Li J, Ma N, Li B (2015). In vivo and in vitro metabolism of aspirin eugenol ester in dog by liquid chromatography tandem mass spectrometry. Biomed Chromatogr.

[14] Li J, Kong X, Li X, Yang Y, Zhang J (2013). Genotoxic evaluation of aspirin eugenol ester using the Ames test and the mouse bone marrow micronucleus assay. Food Chem Toxicol.

[15] Li J, Yu Y, Yang Y, Liu X, Zhang J, Li B, Zhou X, Niu J, Wei X, Liu Z (2012). A 15-day oral dose toxicity study of aspirin eugenol ester in Wistar rats. Food Chem Toxicol.

[16] Karam I, Ma N, Liu XW, Li SH, Kong XJ, Li JY, Yang YJ (2015). Regulation effect of aspirin eugenol Ester on blood lipids in Wistar rats with hyperlipidemia. BMC Vet Res.

[17] Karam I, Ma N, Liu XW, Kong XJ, Zhao XL, Yang YJ, Li JY (2016). Lowering effects of aspirin eugenol ester on blood lipids in rats with high fat diet. Lipids Health Dis.

[18] Ma N, Karam I, Liu XW, Kong XJ, Qin Z, Li SH, Jiao ZH, Dong PC, Yang YJ, Li JY (2017). UPLC-Q-TOF/MS-based urine and plasma metabonomics study on the ameliorative effects of aspirin eugenol ester in hyperlipidemia rats. Toxicol Appl Pharmacol.

[19] Ma N, Liu X, Kong X, Li S, Jiao Z, Qin Z, Dong P, Yang Y, Li J (2017). Feces and liver tissue metabonomics studies on the regulatory effect of aspirin eugenol eater in hyperlipidemic rats. Lipids Health Dis.

[20] Zeng H, Grapov D, Jackson MI, Fahrmann J, Fiehn O, Combs GF (2015). Integrating multiple analytical datasets to compare metabolite profiles of mouse colonic-cecal contents and feces. Meta.

[21] Tian Y, Zhang L, Wang Y, Tang H (2012). Age-related topographical metabolic signatures for the rat gastrointestinal contents. J Proteome Res.

[22] Eckburg PB, Bik EM, Bernstein CN, Purdom E, Dethlefsen L, Sargent M, Gill SR, Nelson KE, Relman DA (2005). Diversity of the human intestinal microbial flora. Science.

[23] Pauwels J, Taminiau B, Janssens GP, De Beenhouwer M, Delhalle L, Daube G, Coopman F (2015). Cecal drop reflects the chickens' cecal microbiome, fecal drop does not. J Microbiol Methods.

[24] Li M, Shu X, Xu H, Zhang C, Yang L, Zhang L, Ji G (2016). Integrative analysis of metabolome and gut microbiota in diet-induced hyperlipidemic rats treated with berberine compounds. J Transl Med.

[25] Lin YC, Yang CC, Chen YJ, Peng WC, Li CY, Hwu CM. Utilization of statins and aspirin among patients with diabetes and hyperlipidemia: Taiwan, 1998-2006. J Chin Med Assoc. 2012;75(11):567–72. 10.1016/j.jcma.2012.08.020 23158034

[26] Venkadeswaran K, Muralidharan AR, Annadurai T, Ruban VV, Sundararajan M, Anandhi R, Thomas PA, Geraldine P (2014). Antihypercholesterolemic and antioxidative potential of an extract of the plant, Piper betle, and its active constituent, eugenol, in triton WR-1339-induced hypercholesterolemia in experimental rats. Evid Based Complement Alternat Med.

[27] Al-Trad B, Alkhateeb H, Alsmadi W, Al-Zoubi M. Eugenol ameliorates insulin resistance, oxidative stress and inflammation in high fat diet/streptozotocin-induced diabetic rat. Life Sci. 2018. 10.1016/j.lfs.2018.11.034. 10.1016/j.lfs.2018.11.034 30448265

[28] Babaei N, Salamci MU (2015). Personalized drug administration for cancer treatment using model reference adaptive control. J Theor Biol.

[29] Giorgi M, Lebkowska-Wieruszewska B, Lisowski A, Owen H, Poapolathep A, Kim TW, De Vito V (2018). Pharmacokinetic profiles of the active metamizole metabolites after four different routes of administration in healthy dogs. J Vet Pharmacol Ther.

[30] Ma N, Yang Y, Liu X, Kong X, Li S, Qin Z, Jiao Z, Li J (2017). UPLC-Q-TOF/MS-based metabonomic studies on the intervention effects of aspirin eugenol ester in atherosclerosis hamsters. Sci Rep.

[31] Getz GS, Reardon CA (2012). Animal models of atherosclerosis. Arterioscler Thromb Vasc Biol.

[32] Yang Y, Smith DJ, Keating KD, Allison DB, Nagy TR (2014). Variations in body weight, food intake and body composition after long-term high-fat diet feeding in C57BL/6J mice. Obesity (Silver Spring).

[33] Stankovic MN, Mladenovic DR, Duricic I, Sobajic SS, Timic J, Jorgacevic B, Aleksic V, Vucevic DB, Jesic-Vukicevic R, Radosavljevic TS (2014). Time-dependent changes and association between liver free fatty acids, serum lipid profile and histological features in mice model of nonalcoholic fatty liver disease. Arch Med Res.

[34] Ben GA, Ben AKR, Chaaben R, Hammami N, Kammoun M, Paolo PF, El FA, Fki L, Belghith H, Belghith K (2017). Inhibition of key digestive enzymes related to hyperlipidemia and protection of liver-kidney functions by Cystoseira crinita sulphated polysaccharide in high-fat diet-fed rats. Biomed Pharmacother.

[35] Xu QY, Liu YH, Zhang Q, Ma B, Yang ZD, Liu L, Yao D, Cui GB, Sun JJ, Wu ZM (2014). Metabolomic analysis of simvastatin and fenofibrate intervention in high-lipid diet-induced hyperlipidemia rats. Acta Pharmacol Sin.

[36] Devi J, Rajkumar J (2014). Effect of Ambrex (a herbal formulation) on hematological variables in hyperlipidemic rats. Pak J Biol Sci.

[37] Gonzalez J, Donoso W, Diaz N, Albornoz ME, Huilcaman R, Morales E, Moore-Carrasco R (2014). High fat diet induces adhesion of platelets to endothelium in two models of dyslipidemia. J Obes.

[38] Chen X, Wang C, Zhang K, Xie Y, Ji X, Huang H, Yu X (2016). Reduced femoral bone mass in both diet-induced and genetic hyperlipidemia mice. Bone.

[39] Dekker MJ, Baker C, Naples M, Samsoondar J, Zhang R, Qiu W, Sacco J, Adeli K (2013). Inhibition of sphingolipid synthesis improves dyslipidemia in the diet-induced hamster model of insulin resistance: evidence for the role of sphingosine and sphinganine in hepatic VLDL-apoB100 overproduction. Atherosclerosis.

[40] Kasbi-Chadli F, Ferchaud-Roucher V, Krempf M, Ouguerram K (2016). Direct and maternal n-3 long-chain polyunsaturated fatty acid supplementation improved triglyceridemia and glycemia through the regulation of hepatic and muscle sphingolipid synthesis in offspring hamsters fed a high-fat diet. Eur J Nutr.

[41] Zhang Y, Wang Z, Jin G, Yang X, Zhou H (2017). Regulating dyslipidemia effect of polysaccharides from Pleurotus ostreatus on fat-emulsion-induced hyperlipidemia rats. Int J Biol Macromol.

[42] Liu YT, Peng JB, Jia HM, Cai DY, Zhang HW, Yu CY, Zou ZM (2014). UPLC-Q/TOF MS standardized Chinese formula Xin-Ke-Shu for the treatment of atherosclerosis in a rabbit model. Phytomedicine.

[43] Jia P, Wang S, Xiao C, Yang L, Chen Y, Jiang W, Zheng X, Zhao G, Zang W, Zheng X (2016). The anti-atherosclerotic effect of tanshinol borneol ester using fecal metabolomics based on liquid chromatography-mass spectrometry. Analyst.

[44] Park HM, Park KT, Park EC, Kim SI, Choi MS, Liu KH, Lee CH (2017). Mass spectrometry-based metabolomic and lipidomic analyses of the effects of dietary platycodon grandiflorum on liver and serum of obese mice under a high-fat diet. Nutrients.

[45] Hong YS, Ahn YT, Park JC, Lee JH, Lee H, Huh CS, Kim DH, Ryu DH, Hwang GS (2010). 1H NMR-based metabonomic assessment of probiotic effects in a colitis mouse model. Arch Pharm Res.

[46] Polakof S, Diaz-Rubio ME, Dardevet D, Martin JF, Pujos-Guillot E, Scalbert A, Sebedio JL, Mazur A, Comte B (2013). Resistant starch intake partly restores metabolic and inflammatory alterations in the liver of high-fat-diet-fed rats. J Nutr Biochem.

[47] Devillard E, McIntosh FM, Paillard D, Thomas NA, Shingfield KJ, Wallace RJ (2009). Differences between human subjects in the composition of the faecal bacterial community and faecal metabolism of linoleic acid. Microbiology.

[48] Zhao L (2013). The gut microbiota and obesity: from correlation to causality. Nat Rev Microbiol.

[49] Mathur R, Kim G, Morales W, Sung J, Rooks E, Pokkunuri V, Weitsman S, Barlow GM, Chang C, Pimentel M (2013). Intestinal Methanobrevibacter smithii but not total bacteria is related to diet-induced weight gain in rats. Obesity (Silver Spring).

[50] Henning SM, Yang J, Hsu M, Lee RP, Grojean EM, Ly A, Tseng CH, Heber D, Li Z. Decaffeinated green and black tea polyphenols decrease weight gain and alter microbiome populations and function in diet-induced obese mice. Eur J Nutr. 2017. 10.1007/s00394-017-1542-8. 10.1007/s00394-017-1542-8 PMC7367598 28965248

[51] Slavov E, Georgiev IP, Dzhelebov P, Kanelov I, Andonova M, Mircheva GT, Dimitrova S (2010). High-fat feeding and Staphylococcus intermedius infection impair beta cell function and insulin sensitivity in mongrel dogs. Vet Res Commun.

[52] Bai J, Zheng S, Jiang D, Han T, Li Y, Zhang Y, Liu W, Cao Y (2015). Hu Y. oxidative stress contributes to abnormal glucose metabolism and insulin sensitivity in two hyperlipidemia models. Int J Clin Exp Pathol.

[53] Lin H, An Y, Hao F, Wang Y, Tang H (2016). Correlations of fecal metabonomic and microbiomic changes induced by high-fat diet in the pre-obesity state. Sci Rep.

[54] Fabian D, Sabol M, Domaracka K, Bujnakova D (2006). Essential oils--their antimicrobial activity against Escherichia coli and effect on intestinal cell viability. Toxicol in Vitro.

[55] He M, Du M, Fan M, Bian Z (2007). In vitro activity of eugenol against Candida albicans biofilms. Mycopathologia.

[56] Zhou X, Rong Q, Xu M, Zhang Y, Dong Q, Xiao Y, Liu Q, Chen H, Yang X, Yu K (2017). Safety pharmacology and subchronic toxicity of jinqing granules in rats. BMC Vet Res.

