# Supplementary material for: Aspirin eugenol ester regulates cecal contents metabolomic profile and microbiota in an animal model of hyperlipidemia
Source: BMC Vet Res. 2018 Dec 18;14:405. doi: 10.1186/s12917-018-1711-x (PMC6299661; doi:10.1186/s12917-018-1711-x)
Supplement: Supplementary file 1 — Table S1. Optimized gradient elution program of UPLC-Q-TOF/MS in cecal content metabonomic study. Table S2. OTU table summary of the samples. Table S3. Difference in relative abundanceof gut microbiotaatphylum level. Figure S1. TIC of cecal content samples in positive and negative modes. Figure S2. PCA score plot basedon the cecal content metabolic profiling in positive and negative modes. Figure S3. Rarefactioncurve of the cecal content samples. (PDF 336 kb) [file 12917_2018_1711_MOESM1_ESM.pdf]

Table S1 Optimized gradient elution program of UPLC-Q-TOF/MS in cecal content metabonomic study.

Time (min)	Mobile phase A (%)	Mobile phase B (%)
0	98	2
2	98	2
7	70	30
20	2	98
22	2	98
23	98	2
25	98	2

Mobile phase A: water with 0.1% formic acid (by volume); Mobile phase B: acetonitrile with 0.1% formic acid.

Table S2 OTU table summary of the samples.

Group	Sample ID	Clean Tags	Effective Tags	OTU numbers
Control	C1	79,575	78,195	866
	C2	83,508	82,101	851
	C4	85,804	84,249	829
	C5	85,068	83,626	792
	C6	89,283	87,789	728
	C7	89,126	87,664	791
	C8	92,267	90,597	908
	C9	79,795	78,464	742
	C10	77,914	76,613	765
	C11	85,566	84,094	841
	C12	81,121	79,617	835
	C13	79,399	78,091	930
	C14	86,489	85,082	895
	C15	79,350	77,953	837
Model	M2	58,188	57,261	684
	M3	61,417	60,345	696
	M4	58,232	57,309	737
	M5	60,640	59,601	694
	M6	65,206	64,126	715
	M7	66,257	65,032	582
	M8	61,407	60,219	612
	M9	61,293	60,281	720
	M10	57,756	56,778	696
	M11	60,316	59,298	651
	M12	54,997	54,211	472
	M13	56,128	55,195	685
	M14	56,496	55,532	639
	M15	63,183	62,175	588

AEE	AEE1	64,912	63,016	676
	AEE2	57,064	55,527	714
	AEE3	65,147	61,384	625
	AEE4	66,236	62,808	649
	AEE5	60,548	59,198	604
	AEE6	64,632	62,981	646
	AEE7	80,289	78,815	757
	AEE8	95,244	91,923	789
	AEE9	94,633	92,571	740
	AEE11	78,064	76,426	622
	AEE12	92,524	90,735	644
	AEE13	85,537	83,840	660
	AEE15	88,921	86,508	625

Table S3 Difference in relative abundance of gut microbiota at phylum level.

Phylum	Control	Model	AEE
Firmicutes	91.11 ±3.76	96.49 ±1.17**	95.72 ±1.31**
Euryarchaeota	3.41 ±2.81	0.20 ±0.13**	0.28 ±0.18**
Actinobacteria	3.31 ±1.63	1.79 ±0.54*	2.34 ±1.04
Proteobacteria	0.33 ±0.25	0.29 ±0.15	0.46 ±0.69
Verrucomicrobia	0.19 ±0.54	0.01 ±0.01	0.01 ±0.01
Spirochaetes	0.04 ±0.02	0.36 ±0.48	0.42 ±0.47*
Tenericutes	0.61 ±0.20	0.44 ±0.15*	0.39 ±0.17**
Bacteroidetes	0.40 ±0.33	0.19 ±0.12	0.23 ±0.15
Saccharibacteria	0.33 ±0.19	0.03 ±0.03**	0.02 ±0.02**
Cyanobacteria	0.08 ±0.08	0.07 ±0.12	0.03 ±0.02
Others	0.18 ±0.05	0.12 ±0.05**	0.09 ±0.03**

Data were expressed as mean ± SD and analyzed by one-way ANOVA with LSD test.
$^{*}P < 0.05$, $^{**}P < 0.01$ compared with the control group.

Figure S1 TIC of cecal content samples in positive and negative modes.

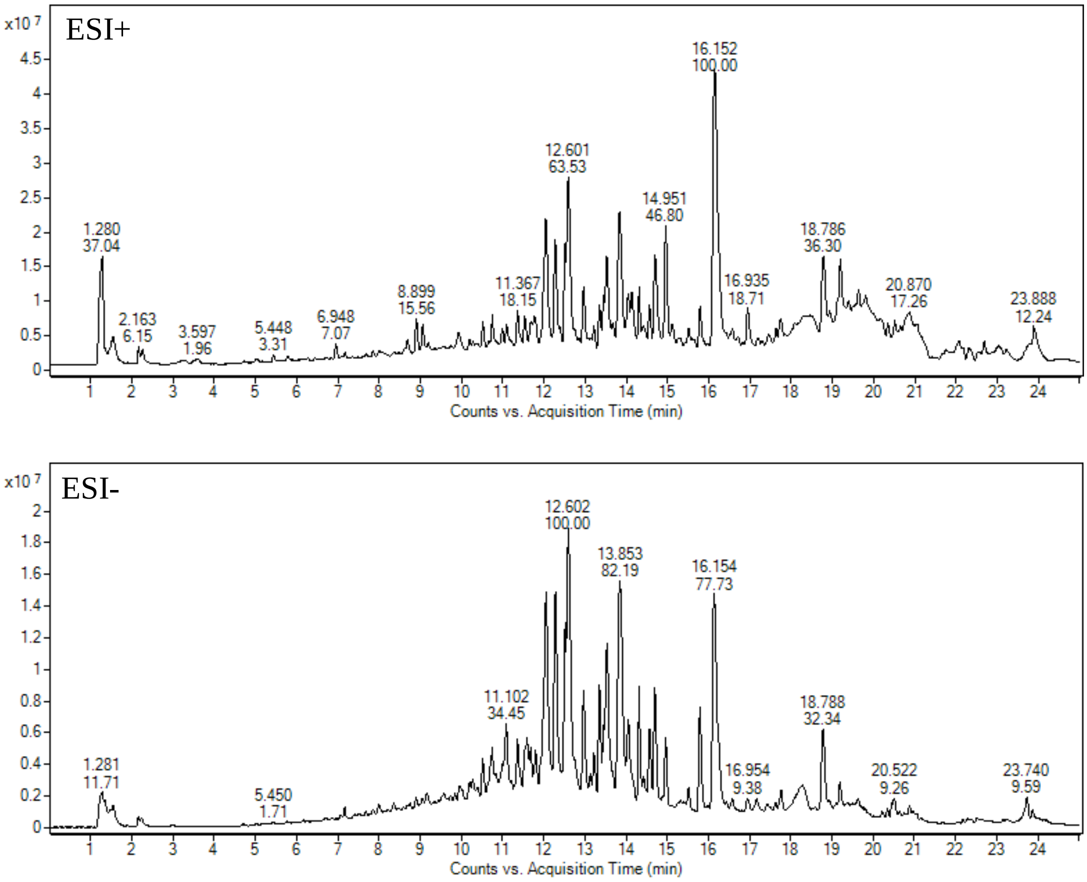

ESI+: electrospray ionization in positive ion mode; ESI-: electrospray ionization in negative ion mode. Retention time and relative peak intensity were labeled on the peaks.

Figure S2 PCA score plot based on the cecal content metabolic profiling in positive and negative modes.

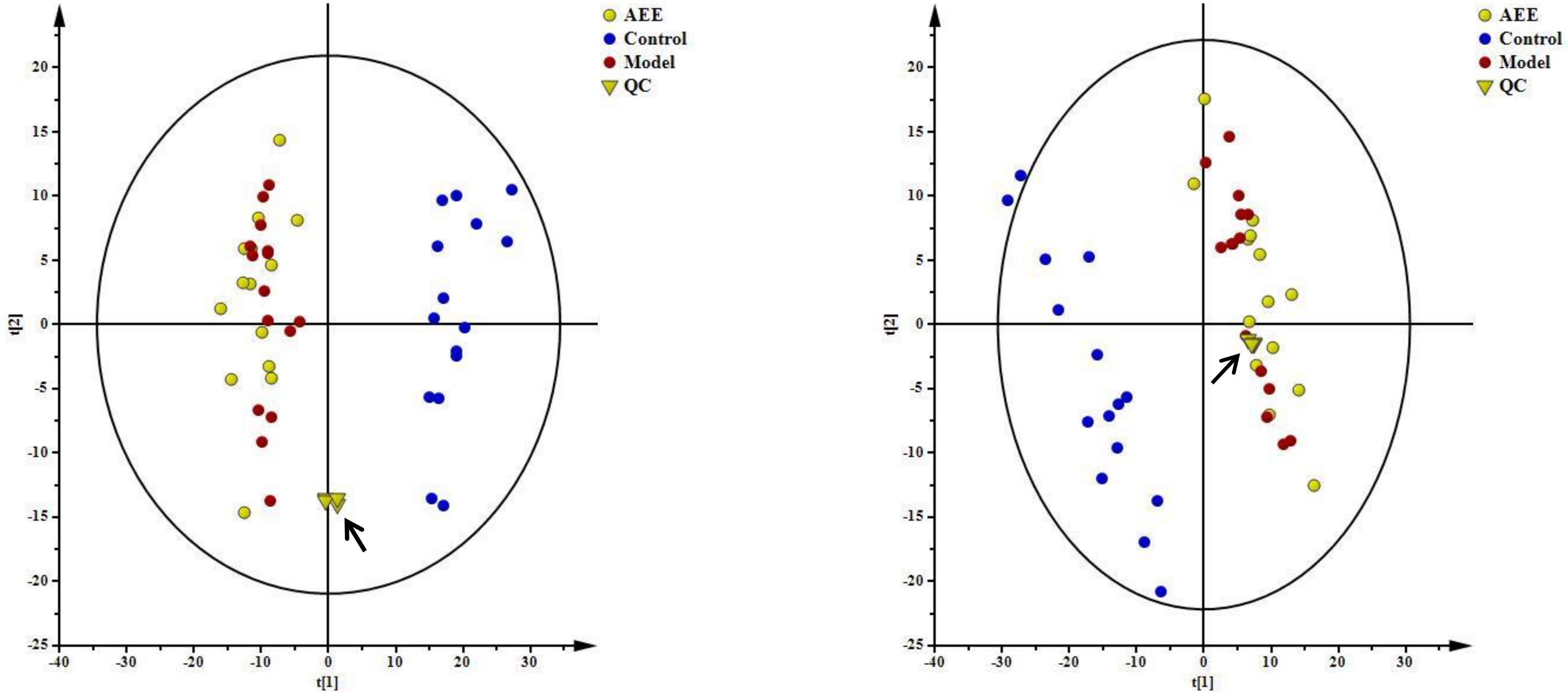

Four QC samples (arrow pointed) were clustered tightly indicating the reliability and repeatability of the data acquisition method.

Figure S3 Rarefaction curve of the cecal content samples

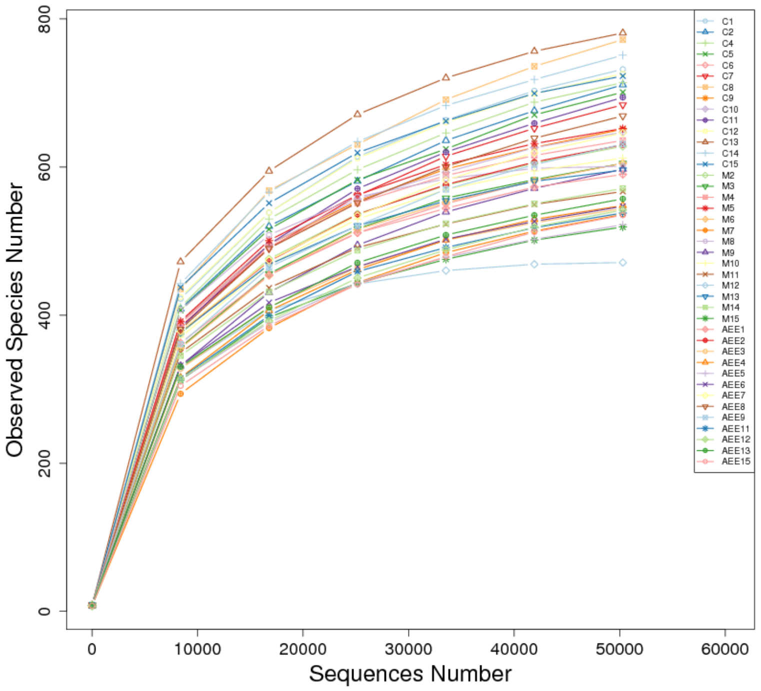

The species numbers gradually increased with the increase of the number of measured sequences and the curve became gentle and the increasing trend became smaller. The rarefaction curve showed that the obtained sequence can reflect the abundance and diversity of the gut microbiota.
